# From Mao to McDonaldization? Assessing the rationalisation of health care in China

**DOI:** 10.1111/1467-9566.13351

**Published:** 2021-08-12

**Authors:** Ross Millar

**Affiliations:** ^1^ Health Services Management Centre University of Birmingham Birmingham UK

**Keywords:** bureaucracy, consumption, health policy, health service organisations, policy analysis, Weber

## Abstract

China's 2009 health care reform agenda has been referred to as one of the most ambitious health policy programmes in modern history. Significant investment has combined with new structures, incentives, and regulations that have aimed to improve access, as well as gain greater control over a health care market much criticised for putting profit before patients. A range of health services research has been undertaken to analyse these efforts. Sociological perspectives have also been documented yet up to now a review and synthesis combining these various contributions has not been undertaken. By drawing on the lens of McDonaldization, the paper presents a narrative review that analyses the extent to which China's 2009 reform agenda has increased efficiency, calculability, predictability, and control over service provision. The review identifies elements of McDonaldization within China's 2009 reform agenda, however, notable gaps remain. In response to the limits of McDonaldization as a lens for understanding China's health care reform, the paper calls for alternative perspectives that are better able to understand the sociocultural dynamics shaping service provision, as well as an interdisciplinary research agenda that is able to generate new insights and understanding regarding health care in China.

## INTRODUCTION

China's health care provision continues to generate a range of scholarly interest (Blumenthal & Hsiao, 2015; Murphy, 2018; Ringen & Ngok, 2017). Outside of the spotlight surrounding its role in the COVID‐19 pandemic (Liu et al., 2020), historical accounts of this vast and diverse country capture how government intervention has struggled to reform the ‘unbridled marketization’ that has enveloped the system since the Reform and Opening Up era (Millar et al., 2016; Yip & Hsiao, 2015). A crucible for such discussion and debate has been China's landmark 2009 health care reforms. These set out to achieve the goal of universal health care provision by 2020 and have been widely hailed for increasing access and affordability to services. Nevertheless, concerns continue to be raised regarding how far the agenda has improved the quality of care and reduced overall costs (Meng et al., 2019; World Bank & WHO, 2016; Yip et al., 2019), with China struggling to shift the ‘entrenched and deep rooted market culture’ and ‘for profit’ ethos guiding service provision (Hsiao et al., 2017; Yi et al., 2017, p. 19).

The 2009 reform agenda symbolised an attempt by the Chinese government to promote access and affordability to services through the implementation of new structures, incentives, controls, and regulations over the health care market. An array of health services research has been undertaken to analyse the impact and effectiveness of these efforts. Sociological perspectives have also been recognised yet up to now a review and synthesis of these various contributions has not been undertaken. This narrative review seeks to draw these various contributions together with a particular focus on the governing rationalities of health care reform in China. Drawing on Ritzer's McDonaldization thesis, the paper analyses the extent to which China's 2009 reform efforts have been able to increase efficiency, calculability, predictability, and control over health care services. The paper will argue that McDonaldization provides a range of explanatory insights into the formal rationalities underpinning China's health care reform efforts, however notable gaps remain with the need for interdisciplinary perspectives to better understand the nature and experience of health care in China.

## FROM MAO TO MARKET: ANALYSING CHINA'S HEALTH CARE REFORM

A range of contributions have documented the ideological and institutional changes that have taken place in China since the founding of the People's Republic in 1949. Often these begin with the arrival of the Mao led government and the implementation of communist planning and prevention policies. The agenda is widely interpreted as a success where an approach rooted in primary and preventative care leads to transformations in the nation's public health. China's ‘barefoot doctor’ primary care model becomes revered and cited as an example of best practice in the Alma Ata declaration, although attacks on the medical establishment, the decline of the hospital sector, and mass starvation are also documented during this period (Blumenthal & Hsaio, 2015; Hesketh & Wei, 1997; Luk, 2017).

The Reform and Opening Up era from 1978 marks a paradigm shift towards the marketisation of health care provision (Wong, 2015; Yip & Hsiao, 2014). China's approach is comparable to other Asian transitional economies during this period, with significant reductions in Government funding and priorities given to economic growth and productivity. However, China's approach also departs from other contexts with its particular use of financial incentives that encourage professionals to increase hospital profits through service fees and bonus schemes (He & Meng, 2015). Concerns are raised during the Reform and Opening‐up period about the ill effects of China's ‘commercialized and market oriented health system’ with the terms ‘kanbing nan’ (expensive access to care) and ‘kanbing gui’ (medical impoverishment) used to describe public discontent (Duckett, 2011; Lin & Zhao, 2015).

**FIGURE 1:**
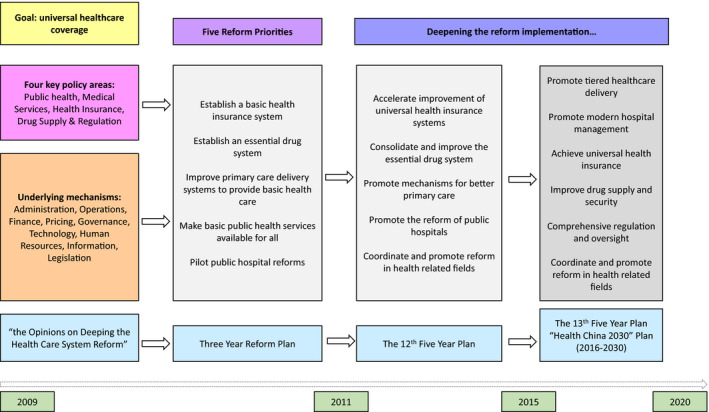
Overall framework and road map of China's health‐care reform agenda (adapted from Tao et al., 2020, p. 3) [Colour figure can be viewed at wileyonlinelibrary.com]

The declaration in 2006 for a ‘socialist harmonious society’ is widely seen as decisive move away from the market‐driven approach towards a greater emphasis on equity and government intervention (Yip & Hsaio, 2015). The change in direction lays the foundations for the 2009 reform agenda (see Figure 1) promoting universal health care provision through a reduction in out‐of‐pocket payments, the expansion of social insurance coverage, greater financial investment in primary care, and improved regulation of professionals and providers (Barber et al., 2014; Lin & Zhao, 2015; Wong, 2015; Yip & Hsaio, 2015).

A range of health services research has sought to capture the effectiveness of these reform efforts (e.g. Meng et al., 2019; Tao et al., 2020; Yip et al., 2019). Calls have also been made for greater understanding of the sociological dimensions of China's reform agenda. Reflecting on the 2009 reform programme at the halfway stage, He and Meng (2015) note that much of the focus and approach taken to understand health care reform in China has been based on the tradition of health economics. Such a viewpoint is echoed by Duckett (2011) noting that health services research has tended to neglect the underlying causes of China's marketised approach with limited considerations given to the commercialisation and privatisation of health care.

Despite these criticisms, interdisciplinary perspectives of China's reform efforts have been captured (He & Meng, 2015). These have included research into the discretionary spaces within policy implementation (He, 2018; Ratigan, 2015), as well as the historical institutional constraints and trajectories of reform implementation (Luk, 2017). Ideological tensions have also been captured, noting that contrary to China's commitment to universal provision as a means to counter the effects of marketisation, the reform agenda has been underpinned by a neoliberal promotion of private capital, a preference for social insurance means testing, and continuing high out‐of‐pocket payments for the population (Zhang & Navarro, 2014). To make sense of these various contributions, the following section argues that China's highly marketised health care landscape has generated what appear to be a unique series of governing rationalities over health care provision. To do so, the section presents how Ritzer's McDonaldization thesis can enable the analysis of health care rationalisation and frame a narrative review of evidence regarding China's 2009 health care reform agenda.

## REVIEWING THE MCDONALDIZATION OF CHINA'S 2009 REFORMS

**FIGURE 2:**
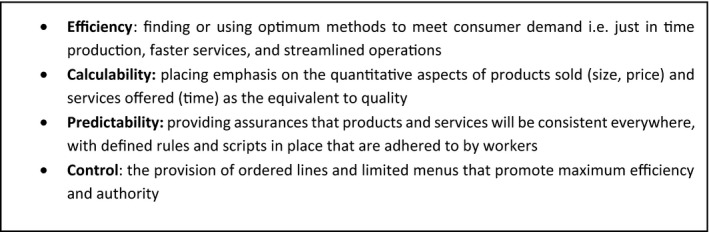
The organising principles of McDonaldization (Ritzer, 2018)

The concept of ‘McDonaldization’ seeks to capture and understand how societies are increasingly subject to greater forms for rational organisation and control (Ritzer, 2018; Waring & Bishop, 2015). The thesis originates from the work of George Ritzer who, inspired by the Weberian belief that societies are increasingly subject to formal rationalities, argued that the organising processes and practices of high speed, large volume, and low price are coming to dominate more and more sectors of society (Ritzer & Miles, 2019). Ritzer characterises McDonaldization across a series of organising principles associated with greater efficiency, predictability, calculability, and control (see Figure 2). These principles can combine to create rationalised cages of consumption and prosumption but also have the potential to generate irrationalities in the form of ‘red tape’ and dehumanising effects associated with poor quality work (Ritzer, 2018).

Applications of McDonaldization in health care have captured the erosion of professional autonomy and the bureaucratisation of work (e.g. Archer et al., 2017; Dorsey & Ritzer, 2016; Ritzer & Walcak 1988; Waring & Bishop, 2013, 2015). In their review of health care McDonaldization, Waring and Bishop (2015) argue that worked examples have much to offer in highlighting the growth of hierarchical control over resource allocation, the horizontal control gained through clinical guidelines and care protocols, and the dynamics of clinical and patient interactions within such contexts.

While McDonaldization has been promoted as a lens to further understand the rationalisation of health care contexts, notable criticisms have been lodged at the approach. Smart (1999) notes the scant attention that the McDonaldization perspective gives to the strategies sustaining the spread of formal rationality, that is, ‘the relentless pursuit of capital accumulation’ (Smart, 1999, p. 4). McDonaldization is susceptible to narrow definitions of consumer behaviour driven exclusively by aspirations for greater convenience and efficiency, with little consideration given to the seduction of consumption itself, or indeed those who criticise and resist the risks and false comfort such consumerism brings (Smart, 1999). Turner (2003) also highlights important limitations in the Fordist logic underpinning McDonaldization where Weberian rationalisation has downplayed the ‘differentiation within modernity’ as local customs and cultures shape the development of McDonaldization processes.

The purpose of the following section is to present a narrative review regarding how far China's 2009 reform agenda has been able introduce greater McDonaldization over service provision. Building on previous research exposure in the area (Millar et al., 2016, 2017; Zhang et al., 2017), electronic searches of Google Scholar between 2019 and 2020 traced theoretical and empirical contributions to the area. Key word searches focused particularly on China's 2009 reform priorities with the terms China and health care reform followed by the additional search terms of ‘health insurance’; ‘primary care’; ‘public health’; ‘public hospital’; and ‘essential medicines’. The review included papers published in English that were deemed relevant based on their comprehensiveness in engaging with the reform priorities, their ability to unearth key tensions and challenges within the reform approach, their overall scholarly contribution in synthesising and generating new insights, as well as any sociological dimensions associated with the ideological, cultural, and socioeconomic consequences of reform. A subsequent snowballing for empirical papers was carried out in May 2021 with electronic searching of PubMed, Web of Science, and Google Scholar using the same search terms in order to update and clarify the key issues and dynamics associated with the various reform elements. A total of 68 articles were included in the review.

The review made sense of the storylines within China's 2009 reform priorities by drawing on the McDonaldization domains as meta‐narratives to bring together to various ways the reforms have been explained and investigated (Greenhalgh et al., 2005). The following sections present these meta‐narratives with a resulting synthesis that aims to surface the rationalities and irrationalities associated with the McDonaldization domains.

## IMPROVING EFFICIENCY

Improving the efficiency of China's health care provision underpinned many of the reform priorities to improve accessibility and affordability of services. The review finds that the promotion of optimal methods to meet population demand for service provision can be identified in government investment to expand social insurance schemes, improve access to public health services, and encourage experiments with alternative provider delivery options.

The expansion of social insurance coverage to 95% of the population is widely hailed in this regard with China Family Panel Studies survey data showing a decline in catastrophic health expenditure for lower socioeconomic groups in particular (Yip et al., 2019). In their review of China's reform goal to promote an equitable and accessible public health service system, Wang et al. (2019) document the significant improvements made in the nine years since the launch of the National Basic Public Health Service Program with regards to population health, where China has seen infant and maternal mortality rates decrease year by year from 2009 to 2016.

While such improvements are documented, the affordability of health care remains a contentious issue with alternative survey data presenting evidence of households struggling to afford the out‐of‐pocket payment charges (Wong, 2015; Yang & Wu, 2014; Zhang et al., 2015). Analysing data from the Chinese Health and Retirement Longitudinal Study survey, Tan et al. (2019) compared the mean costs incurred for both inpatient and outpatient care under different health insurance schemes. These findings show that the expansion of social health insurance (SHI) coverage did not lead to a significant reduction in out‐of‐pocket (OOP) payments for outpatient care compared to those uninsured. To explain these findings, other research draws attention to how expanded insurance coverage has often been constrained by a fee‐for‐service payment structure that incentivises hospitals to increase rather than control their costs (Tian et al., 2015; Yip & Hsiao, 2014). Wang et al. (2019) note how the oversupply of services and overprescribing of treatments remain established norms to generate additional revenues for providers. Inequalities are also noted with insurance coverage privileging urban over rural populations (Yip et al., 2019), and rural migrants to urban areas remaining unable to access health care services (Luk, 2017).

China's reform efforts to improve access to primary and community care have reported positive patient and public experiences associated with these new facilities (McCollum et al., 2014). However, such benefits are overshadowed by continuing concerns regarding the quality and capability of services (Li & Yu, 2011; Liu et al., 2014; Wei et al., 2015). Outpatient attendance data are used to demonstrate how primary care has struggled to perform a gate‐keeping function with the new infrastructure failing to attract patients compared to tertiary hospitals (Yip et al., 2019; Zhang et al., 2011). Low use and capacity of primary health care is also explained by the embedded fee‐for‐service payment system, which has incentivised hospitals to attract and retain patients who could otherwise use primary health care providers (Meng et al., 2019). The hospital centred system has raised concerns about how far China can meet the non‐communicable disease (NCD) challenge the country faces (Li et al., 2017; Yip et al., 2019) with limited consideration given to social and environmental factors such as health promotion and environmental pollution (Wang et al., 2020).

China's experimentation with other delivery options to improve access has also included greater investment in internet technology and options for promoting integrated care (Yip et al., 2019). These interventions report improved experiences, reduced costs, and improved accountability systems from case study examples such as the ‘Sanming model’ (Fu et al., 2017). However, these have up to now had limited uptake system wide due to a lack of incentives and supporting infrastructure (He & Yang, 2015; Yip et al., 2012). In their review of public hospital reforms, Mei and Kirkpatrick (2019) suggest that plans to strength their public orientation and empower management are likely to struggle given the bureaucratic culture of public administration and party hegemony over government official involvement within hospital governance structures. Other provider reform options in the literature capture the drive to increase private sector involvement within the hospital sector. Descriptive statistical analysis by Pan et al. (2016) reports that increased hospital competition correlated with higher outpatient quality and lower outpatient medical costs. Yet the private hospital sector (measured by hospital beds) has struggled to compete with the public hospital sector which accounts for nearly 90% of the market share.

## CONTROLLING CALCULABILITY

The logic of calculability—defined as the quantitative aspects of products sold and services offered—has long been a feature of China's health care system where the extensive use of quantity based bonuses has led to the provision of unnecessary care and the crowding out of intrinsic motivations. To quell the need to generate profits from fee for services, the review captures how the 2009 reforms encouraged local governments to experiment with alternative payment methods such as global budgets, diagnosis‐related groups, and capitation payments. These alternative payment methods have been associated with notable improvements such as reported decreases in total medical expenditures, out‐of‐pocket costs, and reduced length of stay (He & Meng, 2015; Pan et al., 2016; Powell‐Jackson et al., 2015; Zhou et al., 2015; Zou et al., 2018). Jian et al. (2015) found that the implementation of pilot Diagnosis Resource Group (DRG) payments in Beijing led to significant reductions in expenditures and out‐of‐pocket payments per admission. However, while no significant cost shifting was observed, the authors also found that ‘loose implementation’ of the DRG payment reform allowed hospitals to divert patients who were older or had more complications, and therefore more expensive to treat, to the fee‐for‐service system. Such findings echo the cost shifting tactics that have been observed outside of the alternative payment structures with increased costs passed on to uninsured patients to compensate for reduced revenues from insured patients (Wang et al., 2011; Zhang, 2010).

The implementation of alternative payment mechanisms have also raised concerns about the lack of quality assurance and monitoring mechanisms in place (Zou et al., 2018). Jian et al. (2019) note that the introduction of DRG payment mechanisms did not lead to the desired quality improvements. Rather, these authors suggest that the motivation for cost reductions in a DRG payment may be driving reductions in the prescription of critical medications and negatively impacting on the quality of care. Relationships between quality of care and existing payment methods also resonate with a range of findings from workforce experience surveys. These chart what appear to be low job satisfaction and morale across the health care workforce, but particularly those working in primary care due to excessive workload, poor salaries, and limited career development (Kong & Yang, 2015; Wu et al., 2014; Xiao et al., 2014; Zhang et al., 2015, 2017; Zhou et al., 2014).

In the absence of other performance information, research draws attention to how patient volume represents a primary measure of hospital performance with a larger hospital sizes often equated with higher quality services, thus reinforcing self‐referrals to larger hospitals and the drive to achieve large tertiary status (Millar et al., 2017; Pan et al., 2016). The situation has been defined by Qian et al. (2019) as a ‘medical arms race’ with the rapid expansion of tertiary hospitals and the drive for expensive diagnostic services being seen as an opportunistic reaction to compensate for financial shortfalls occurring elsewhere. Within these contexts, technology investment provides a ‘non‐quality indicator’ to attract patients with additional income generated from higher patient volumes (Qian et al., 2019). A systematic review of hospital performance measures by Yu et al. (2019) document how the unevenness of health care resources is closely related to a city's administrative rank and power, with a higher level equating to better resources and a stronger tertiary hospital sector.

## LIMITED CONTROL

China's health reform efforts have sought different ways to control service provision through the introduction of guidelines and essential medicines management. The review finds evidence of ordered lines and limited menus has been particularly evident in China's approach to the regulation of drugs and medicines as well as drives for improved regulatory coordination and oversight.

With drug revenues reported to account for over 40% of total hospital revenues (Yip & Hsaio 2014) and antibiotic abuse described as widespread (Currie et al., 2011), a Zero‐Markup Drug (ZMD) Policy sought to eliminate the 15% mark‐up allowance on prescribed drugs across hospitals and primary care. Reductions in drug expenditures have subseqeuntly been documented (Yip et al., 2019), with an interrupted time series analysis by Liu et al. (2021) showing that the ZMD and the more recently implemented Zero Markup for Consumables policy were associated with reductions in drug revenues for hospital providers and total expenditure for outpatient and inpatient services. Their research indicates that the medical pricing reform successfully restricted the profit‐seeking behaviour in drug and consumable sales. However, Liu et al. (2021) also note unintended consequences resulting from these changes, with significant increases in consultation fees and inpatient activities since the policy changes being attributed with making up the shortfall in losses from drug sales. Research documents how the overuse and misuse of drugs has also continued as doctors fearful of disputes engage in defensive medicine practices (He, 2014; He & Qian, 2016).

The introduction of an essential medicines list (EML) aimed to counter antibiotic abuse, along with escalating costs, by requiring health care providers to stock and prescribe only drugs from the EML (Wong, 2015). The list has also reportedly reduced average costs per prescription and inappropriate use of medicines (He & Meng, 2015; Zhou et al., 2015). Nevertheless, the limited range of drugs has caused problems for frontline workers as they have experienced reductions in income and loss of autonomy in what options they can offer to patients (Zhou et al., 2014).

Despite the drive for greater transparency and regulation over provision, corruption continues to be reported. Mixed methods research by Shi et al. (2018) found that medical corruption remains a concern with root causes attributed to the financial pressure on public hospitals and professionals to generate additional revenue to meet the shortfall in essential medicines list reimbursement and subsides from national and local government. A narrative review by Zhu et al. (2018) looked further at the issue of ‘red envelopes’ containing money which are given as gifts by patients. Described as ‘a commonplace phenomenon in medical practice’, the authors also argue that such practices can be explained as a way to compensate for work pressure and limited incomes, along with the erosion of professional ethics that has taken place over time.

The evidence review by Meng et al. (2019) notes that a central challenge to gaining control over provision has been insufficient coordination and oversight. In 2018, China established the National Healthcare Security Administration (NHSA) to better coordinate insurance programmes and drug procurement. In addition, the National Health Commission (previously the Ministry of Health) also has a role in the planning, administration, and regulation of the health care system. Yet despite government efforts to build ‘health in all policies’, much work is still needed to build cooperation and coordination across government institutions.

## (UN)PREDICTABILITY

The 2009 reform agenda sought to improve the regulation and oversight of health care provision. However, the review findings show that approaches have had a limited impact on predictability in terms of assurances that products and services will be characterised by formalisation, routine, and consistency. An evidence review by Jiang et al. (2015) notes that despite progress with increasing access to services, continuing gaps in the quality of care are rooted in inadequate performance measurement. The launch of the Hospital Quality‐Monitoring System in 2011 aims to cover all tertiary hospitals, however, the measures being used remain limited to length of stay and mortality with inadequate clinical information related to other clinical indicators or quality measures.

Overall patient safety performance remains low compared to other health care systems (Yip et al., 2019). The precarious situation surrounding the predictability of services has been captured in the rationalisation of maternity care services. To explain why China has one of the highest caesarean section rates in the world, an evidence review by Long et al. (2018) identified a range of sociocultural factors that explained the increasing accessibility and acceptability of this procedure. These included poor relationships between women and health professionals based on widely held beliefs about the deteriorating quality of care during birth caused by a lack of labour support and pain relief measures. Financial drivers have also been identified as influencing health professionals’ preference for caesarean section, where performing an elective surgery can generate higher financial benefits for physicians than vaginal delivery.

China's reliance on financial incentives to generate income for organisations has had a detrimental effect on doctor patient relationships (Blumenthal & Hsaio 2015; Cao & Wei, 2014; Gao & Gurd, 2015; Yip et al., 2012). Ethnographic research by Chan (2017) documents how institutional and interpersonal mistrust have been rooted in the perceived injustices of clinical decisions skewed towards maximising income. These dynamics have been overlaid by a societal mistrust that began to emerge from the Cultural Revolution (1966–1976) onwards. The unpredictability of doctor patient encounters has been brought to the fore with a rise of reported disputes and violence against professionals causing physical and psychological harm (He & Qian, 2016; Synder 2015; Zhang et al., 2015, 2017). Such events have been explained by a lack of transparency (Xie & Zhang, 2014) along with limited opportunities for patients to voice their grievances or make complaints (Blumenthal & Hsaio 2015; Snyder, 2015). Patient dissatisfaction has also been associated with an increasing willingness to challenge medical decisions and outcomes (Yu et al., 2015). With ‘unrealistically high expectations’ reported from patients and families (Tucker et al., 2015), further professional training and development has been advocated to improve the communication skills of the health care workforce (Cai et al., 2010; Liu et al., 2014).

## THE MCDONALDIZATION OF CHINA'S HEALTH CARE: RESISTANCE AND CRITIQUE

China's 2009 reform agenda introduced a range of measures to achieve the goal of universal health care provision by 2020. The rapid expansion of health services research during this period has captured a range of insights with statistical analysis and cross‐sectional surveys identifying improved efficiencies resulting from the expansion of social insurance schemes and the investment in public health and primary care provision. Research identifies evidence of increased calculability with the promotion of alternative forms of payment as well as greater predictability and control over service provision through the introduction of clinical guidelines and increasing regulatory oversight.

Qualitative and quantitative research also provide explanatory insights into the limits or ‘irrationalities’ associated with the McDonaldization domains (Table 1). The findings raise a number of tensions between the formal rationalities within China's 2009 reforms and the existing market rationalities that pervade the current health care system (Ritzer & Walczak, 1988). China's experience draws similarities with Light's (1995) depiction of a ‘medical industrial complex’ where corporations, in this case public hospitals, have sought to maximise profits through product innovations and improvements that tap into consumer aspirations for higher quality services.

**TABLE 1:** The McDonaldization of health care Reform in China

Domains of McDonaldization	Reform examples	Consequences and implications
Efficiency	Increasing government investment Expansion of insurance schemes Improving access to primary care Promotion on integrated care options and greater provider diversity	Increased access and equity to services Affordability remains issue due to existing fee for service incentives driving up costs Low uptake of primary care due to quality concerns and hospital dominance who are incentivised to attract and retain patients Limited impact of public hospital reforms as providers lack incentives to change existing routines and practices
Calculability	Alternative payment methods to reduce gaming behaviours of providers incentivised to increase costs	Positive impact on costs and experience however limited overall impact on costs and behaviours as income generated from elsewhere
Control	Zero‐Markup Drug Policy controlling overuse and misuse of prescription drugs Essential Medicines List standardising drug options Improved regulation and coordination of health care institutions	Reductions in expenditures and misuse of drugs Created unintended consequences with increased costs and activities elsewhere Knock on effects for workforce with loss of income and strains on patient relationships
Predictability	Efforts to improve quality assurance with increased performance reporting One stop shop services e.g. maternity	Poor professional‐patient relationships characterised by mistrust

Various recommendations are put forward to resolve the tensions within China's reform agenda. These include a further emphasis on population health and primary care development (Liu & Saltman, 2019; Powell‐Jackson et al., 2015; Yip et al., 2019), as well as greater emphasis on workforce development and wellbeing (Chen et al., 2014; Hung et al., 2013; Tucker et al., 2015; Zhang et al., 2015). To do so, Ramesh et al. (2015) along with others call for increased government intervention in order to hold providers to account for efficiency, quality, and costs (Allen et al., 2014; He & Meng, 2015; Pan et al., 2016; Tian et al., 2015; Wong, 2015; Yip et al., 2019; Yip & Hsiao, 2014). An increase in public health infrastructure as well as greater cooperation, coordination, and communication are also advocated in light of China's experience during the COVID‐19 pandemic (Wu & McGoogan, 2020).

However, given the current trends and trajectories identified by the review, the implementation of any such recommendations remain some way off. Yet while the lens of McDonaldization has offered the review opportunities to gain further critical insights into China's reforms, such an approach is not without limitations. As identified in the earlier critique, McDonaldization is susceptible to downplaying the importance of local cultures and meanings in the translation of these domains (Turner, 2003). Such limitations and concerns have particular relevance to the applicability of Weberian assumptions within a context such as China. Both Lai (2015) and Tian (2003) point out that Weber's understanding of rationality, central to McDonaldization, builds from a view that simultaneously privileges Western capitalism while also simplifies the complexity of historical and cultural traditions not fully understood in Western terms and categories. A Weberian analysis is susceptible to ‘Orientalism’ and ‘Eurocentrism’ in neglecting other facets of China's socio‐historical context, most notably Confucianism and Daoism (Lai, 2015).

Alternative perspectives of sociocultural relevance to China have the potential to generate new insights that might otherwise be missed from McDonaldization. Zou et al. (2018) describe how guanxi—defined as personal connections or interpersonal networks—has long been used as ‘an informal and culturally distinctive strategy’ to facilitate business relations or acquire governmental services, yet the concept has, up to now, rarely featured within the health services research undertaken into China's health care reforms. Zou et al. (2018) capture how guanxi connections can be mobilised by patients in order to gain better medical services. As a result, these authors call for anthropological research into the views and lived experiences of socially and economically disadvantaged groups with no guanxi ties to exploit, as well as the power asymmetries between patients and physicians within guanxi clinical encounters. An area underrepresented by the health services research evidence, but of particular relevance to China, is the role of Traditional Chinese Medicine (TCM). Xu and Xia (2019) chart the rapid expansion over recent years with TCM development in becoming an integral part of China's ‘Belt and Road’ global infrastructure strategy alongside its central role within Chinese society. Given its significance, further research is needed to examine the interactions between TCM and other health care reforms.

To complement the burgeoning health services research that is predominately informed by quantitative and cross‐sectional survey methods, the implications of the review draw attention to the importance of qualitative research in gaining further explanatory insights into the experiences of reform. While the undertaking of in depth, potentially sensitive, research in China poses a number of challenges, a review and synthesis of ethnographic and anthropological studies of health related fields within China would provide valuable insights into the underlying trends and dynamics surfaced by the review. For example, the ethnographic work of Lora‐Wainwright (2010) captures how villagers in rural Sichuan would employ a range of lay aetiologies such as negative emotions, smoking, alcohol, and preserved vegetables to explain the causes of cancer rather than the environmental pollution that has been widely attributed with the rapid rises in cancer rates within such areas. Ethnographic research, therefore, has the potential to shed new insights into the non‐communicable disease challenge facing China. In doing so, such research can respond to the challenges facing China's primary health care system, as well as calls made elsewhere for further research studies into the evolution and socioeconomic environment of primary care in China (Li et al., 2017).

Greater attention to the historical institutional context surrounding China's current health care reform efforts is also needed. Where China's 2009 reform efforts have been connected to a government shift away from marketisation (synonymous with the Reform and Opening Up era), a deeper understanding of the historical institutional dynamics of Maoist period would provide further explanation. Song and Smith (2019) note how current literature on the rural‐urban disparities of health have primarily focused on inequalities created by geographical differences, but with insufficient understanding of how the household registration system or hukou system (first established in 1955) has acted as a ‘form of social control’ to exclude the rural population from access to welfare services. Where rural migrants in urban areas face discrimination and social stigma, Song and Smith (2019) call for further research into how hukou types create inequalities in various aspects of physical and psychological health.

## CONCLUSION

China's 2009 reform agenda introduced significant investment along with a range of structures, incentives, and regulations to achieve universal health care provision and improve quality of care. This review has sought to appraise and synthesise a range of contributions that have analysed how far China has achieved these reform goals. To do so it has employed the domains of McDonaldization to show how the 2009 reforms have gone some way to achieving greater efficiency, calculability, predictability and control over service provision. However the review also identifies clear limits and challenges within these efforts, as they have sought to disrupt the market rationalities that pervade China's health care provision.

The lens of McDonaldization has provided the review with a range of explanatory insights into the governing rationalities of China's health care reform. Such a perspective has the potential to open up debate regarding current approaches to health care reform within China but also more widely across global health care contexts. However, such a perspective is not without its limitations. The review identifies notable omissions and limits with such approaches in being able to explain and understand the sociocultural dynamics and contexts shaping reform activity.

Further research is needed to better understand the implications of these findings. An important limitation of this review has been the exclusion of publications in Chinese. A review led by a United Kingdom researcher is also exposed to omitting key insights and dynamics within the evidence being analysed. Therefore, further systematic reviews of evidence regarding China's health care reform are needed that draw on both English and Chinese databases. An interdisciplinary research agenda is also needed to further understand the issues raised by the review, with further exploration of sociological perspectives of health care reform that have up to now rarely featured in China's health services research agenda.
